# P-989. Development of a multidisciplinary guideline for diabetic foot infections at a Veterans Affairs Hospital

**DOI:** 10.1093/ofid/ofaf695.1188

**Published:** 2026-01-11

**Authors:** Ronald E Kendall, Elizabeth A Scruggs-Wodkowski, Robert Woods, Kathleen A Linder, Shiwei Zhou

**Affiliations:** VA Ann Arbor Healthcare System, Ann Arbor, Michigan; Veteran Affairs Ann Arbor Healthcare System; University of Michigan Medical School, Ann Arbor, Michigan; University of Michigan, Ann Arbor, Michigan; University of Michigan/Ann Arbor VAMC, Ann Arbor, Michigan; Michigan Medicine, Ann Arbor, Michigan

## Abstract

**Background:**

Complications of diabetic foot ulcers (DFUs) are common, often lead to hospital admission, and benefit from a multidisciplinary treatment approach. IDSA guidelines recommend selecting antibiotics for infected DFUs based on likely pathogen(s) and their antibiotic susceptibilities, but regional variation in resistance patterns creates challenges. We created a workflow and novel scoring tool to facilitate diagnostic and therapeutic management of DFUs within the Veterans Affairs Ann Arbor Healthcare System (VAAAHS).

**Figure 1.**
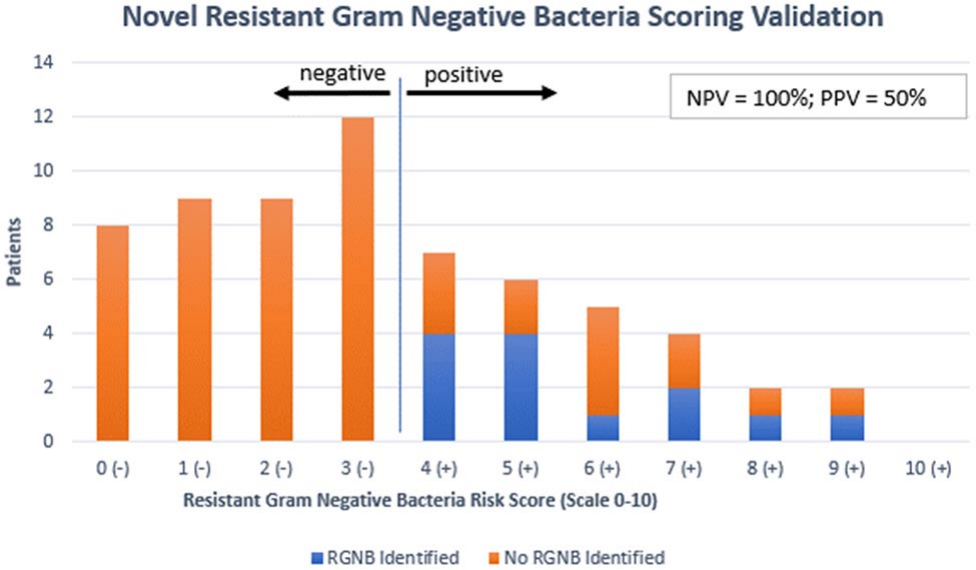
Novel Resistant Gram-Negative Bacteria Scoring Validation Abbreviations: NPV: negative predictive value; PPV: positive predictive value; RGNB: resistant Gram-negative bacteria

**Figure 2.**
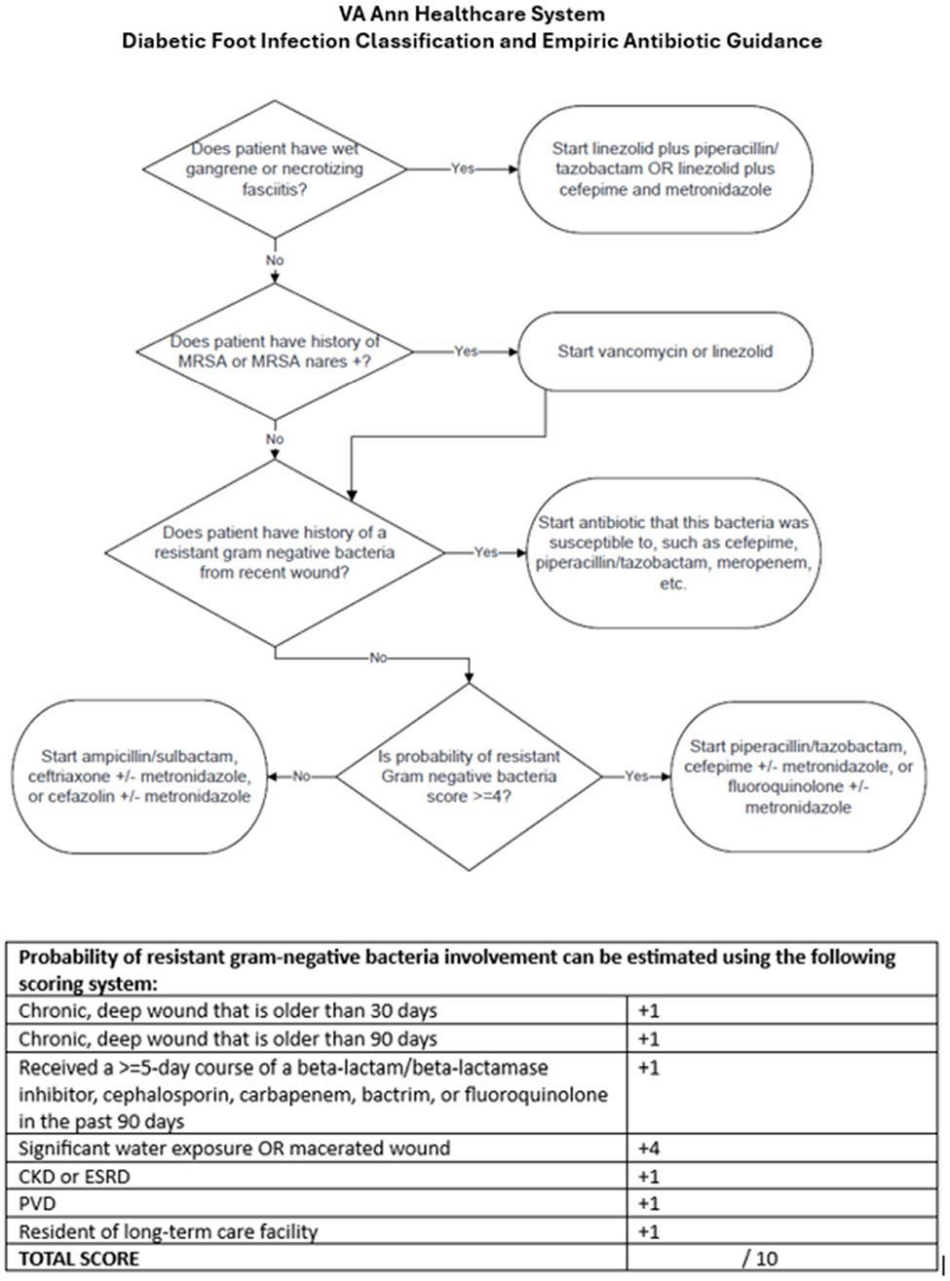
Diabetic Foot Infection Classification and Empiric Antibiotic Guidance Abbreviations: CKD: chronic kidney disease; ESRD: end-stage renal disease; MRSA: methicillin-resistant Staphylococcus aureus; PVD: peripheral vascular disease

**Methods:**

Infectious Diseases, Podiatry, and Surgery clinicians and pharmacists at VAAAHS collaborated to review DFU guidelines, DFU epidemiology, and the local antibiogram to create a DFU workflow as well as a novel scoring tool to assess risk of DFU infection with resistant gram-negative bacteria (the RGNB risk score). RGNB were defined as *Pseudomonas* spp., *Acinetobacter* spp., or Gram-negative organisms resistant to ceftriaxone or with moderate risk for inducible AmpC production. To validate this tool, we performed a retrospective chart review of 78 patients admitted with infected DFU from May 2023 - July 2024. Risk factors were tallied and each case assigned a RGNB risk score between 0-10.

**Figure 3.**
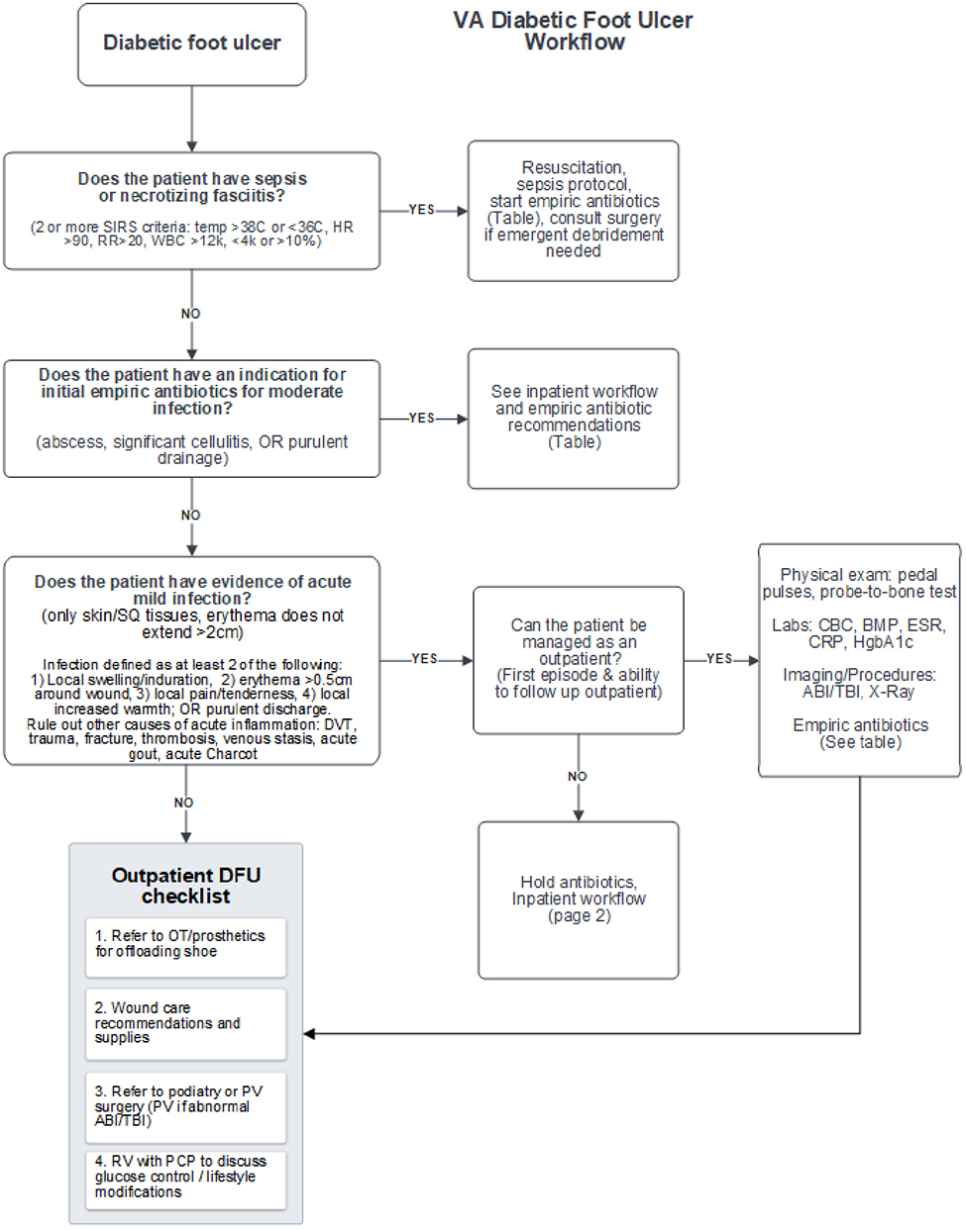
Diabetic Foot Ulcer Workflow Abbreviations: ABI/TBI: ankle brachial index / toe brachial index; DVT: deep vein thrombosis; OT: occupational therapy; PCP: primary care provider; PV surgery: vascular surgery; RV: return visit; SQ: sub-cutaneous

**Figure 4.**
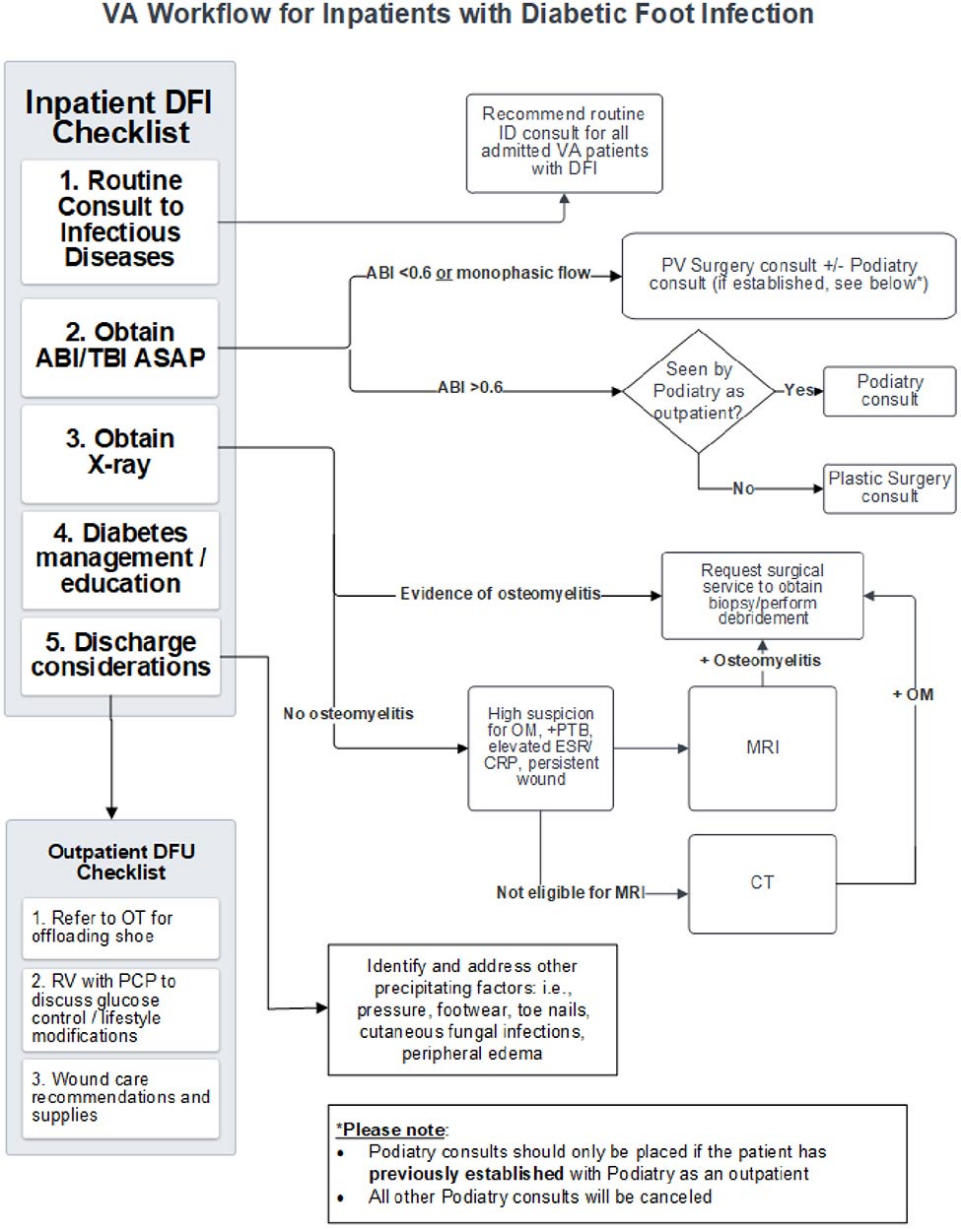
VA Workflow for Inpatients with Diabetic Foot Infection Abbreviations: ABI/TBI: ankle brachial index / toe brachial index; DVT: deep vein thrombosis; OM: osteomyelitis; OT: occupational therapy; PCP: primary care provider; PTB: probe-to-bone; PV surgery: vascular surgery; RV: return visit; SQ: sub-cutaneous; VA: Veterans Affairs

**Results:**

No patients with a score less than 4 had a RGNB in a culture from their infected DFU, indicating 100% negative predictive value (NPV) at this cutoff (Figure 1). We incorporated this scoring tool into an algorithm that included an assessment of gangrenous or necrotizing features, methicillin-resistant *Staphylococcus aureus* (MRSA) screening status, and history of RGNB (Figure 2). Algorithms were designed for outpatient and inpatient management of DFU, with a focus on severity of infection and initial diagnostic workup (Figures 3 and 4).

**Conclusion:**

A dedicated screening tool developed by a multidisciplinary workgroup was designed to assess RGNB risk; it had 100% NPV when the score was < 4. The RGNB risk score may help clinicians make more appropriate empiric antimicrobial decisions for DFU infectious complications, but further study is needed to determine impact on antimicrobial choice and hospital length of stay.

**Disclosures:**

All Authors: No reported disclosures

